# A qualitative exploration on accounts of condom-use negotiation with clients: challenges and predicaments related to sex work among street-based female sex workers in Ekurhuleni District, South Africa

**DOI:** 10.11604/pamj.2021.40.54.29918

**Published:** 2021-09-22

**Authors:** Nokuthula Sikhosana, Mathildah Mpata Mokgatle

**Affiliations:** 1 Department of Epidemiology and Biostatistics, School of Public Health, Sefako Makgatho Health Sciences University, Ga-Rankuwa, 0208, Afrique du Sud

**Keywords:** Female sex worker, condom use, unprotected sex, vaginal health

## Abstract

**Introduction:**

female sex workers (FSWs) are the key vulnerable populations since they carry the high burden of HIV and sexually transmitted infections (STIs). However, the vulnerability of street-based FSWs to HIV/STIs is much higher than that of the establishment-based FSWs. The study aimed to explore street-based FSWs’ condom negotiation skills, barriers to condom use as well as the challenges and predicaments they face on a daily basis.

**Methods:**

an exploratory qualitative approach using focus group discussions was conducted among FSWs working in a major provincial road in a district of Gauteng Province. Thematic content analysis using NVivo version 10 software was conducted.

**Results:**

the age range of the FSWs was 19 to 44 years. The themes that emerged from the data on challenges to negotiation and condom use among FSWs revealed the ways condoms are used in early sex work and over time, ways of enforcing condom used, preferred types of condoms and the predicaments to working in the sex trade. FSWs gained experience of negotiating condom use over time in their work. Both female and male condoms were available and accurate insertion of condoms was reported. Male condom was preferred. Condom use strategies included direct request; using health-information messages; charging more for condomless sex; and refusing condomless sex. The FSW reported the risks of violent attacks of unregulated street-based environment.

**Conclusion:**

condom negotiation strategies illustrated that peer-education and sharing experiences among themselves were beneficial for protective sexual behaviours. Peer-education benefits and peer-interactions yielded assertive attitudes and behaviours of demanding and/or enforcing condom use.

## Introduction

Globally, the large burden of HIV infection is disproportionately distributed among the female sex workers (FSWs) [1-4]. Female sex workers are like other key populations who face stigma, discrimination and criminalization, hence they are marginalized and hidden, resulting in greater risks of HIV acquisition and transmission [1,5-7]. Female sex workers also experience high levels of social and health problems such as unwanted pregnancies, STIs and HIV infections [8,9]. In countries with HIV epidemics, such as South Africa, there is high prevalence of HIV among FSWs in urban areas, with the overall odds of HIV infection being five times higher than in the general female population [10]. Data show that about 4.3% of women engage in sex work in sub-Saharan Africa [11]. Many women, whether married or single, employed or unemployed, as well as young girls between the ages of 13 and 17 years old, enter sex work [12]. According to Jie *et al*. [13] the practice of sex in exchange for money has been reported for centuries, with the majority of FSWs starting to do sex work at a very young age. In most cases, entry into sex work in cities is said to be caused by low literacy levels, and lack of employment opportunities that pay descent remuneration packages [3,14].

The correct and consistent use of condoms during sexual relations is one of the most effective strategies for HIV and STI prevention, hence HIV prevention and promoting condom use is essential [3,14]. FSWs lack power to negotiate condom use, due to their being in unequal relationships with male clients who are their source of income [3,14]. Tamene *et al*. found that FSWs did not use condoms with all of their sexual partners, regardless of whether they were partners, boyfriends, husbands or clients [15]. Non-condom use with steady partners is a great concern, given that the partners may contribute more to FSWs´ STI and HIV risks than clients do, more so that some of the partners were encountered during sex work [3,16]. The point of contact with male clients was also found to be influential with regard to condom use, because street-based FSWs reported less condom use than those working in well-structured environments like hotels and massage parlours. In most cases, street-based FSWs had lower education levels, charged less for sex, and had been sex workers for a longer period than those who were establishment-based, hence the vulnerability for STIs [1].

Numerous countries including South Africa have adopted intervention programs for FSWs that focus on increasing condom use as a strategy, because of the effectiveness of consistent condom use for preventing the acquisition and transmission of HIV/STIs [3,17]. Condom use with a male client depends on the ability of FSWs to effectively negotiate condom use [14]. However, the literature shows that FSWs lack condom use negotiation skills with clients [14]. Furthermore, some FSW´s who are also drug users have low self-esteem due to self-prejudice of being in the sex trade, and therefore feel deprived of their right of choice regarding condom use [8]. The objectives of this study were to explore street-based FSWs´ condom use practices, condom negotiation skills, and the barriers to condom use, as well as the challenges and predicaments they are faced with on a daily basis. The knowledge gained from the study may be used to contribute to the implementation or strengthening of public policies focusing on the importance of condom use by both clients and female sex workers, and to finding ways to reduce vulnerability of street-based FSWs.

## Methods

This study employed an exploratory qualitative approach, which was conducive to investigate the phenomenon studied. This study was a research project for a Masters of Public Health at Sefako Makgatho Health Science University by the first author. The study addressed a range of issues relating to condom use by female sex workers and their clients.

### The study setting and population

Data collection was conducted at the Centre for Positive Care offices (CPC), a non-governmental organisation in Gauteng Province. The Centre is funded by the South African Department of Health and it supports the National Strategic Plan of South Africa to reduce HIV, STIs, and TB, and to strengthen reproductive health rights services. The population for the study consisted of the street-based FSWs working in Heidelberg Road, one of the main urban roads in Gauteng Province. For participant recruitment, the site coordinator at CPC assisted with gaining entry to the FSWs as they were accessing their health care services at the Centre. The Centre provided the researcher with peer-educators who accompanied the researcher to the Heidelberg Road, where the FSWs work, to facilitate recruitment. The purpose of the research was shared with the participants.

Purposive sampling technique was used to select and enrol street-based sex workers into focus group discussions (FGDs), and those interested were asked to reportto the CPC offices for the FGDs. The inclusion criteria for the study were being an adult FSW aged 18 years and above, doing street-based sex work for six months and more. Consenting FSWs were fetched in the morning from their place of work along the identified main road on the days when discussions were due to take place and taken to CPC offices. The study employed focus group discussions as an approach to data collection because the participants for the study shared similar characteristics. Six FGDs consisting of 4 to 11 participants were conducted with a total sample of 46FSWs. Six focus group discussions were conducted, through which data saturation was reached and no new information emerged from the interviews.

**Data collection**: for data collection, the researcher used an interview guide with open-ended questions and a socio-demographic section. Discussion questions included family life and relations, sex work experiences and challenges, health related risks, negotiating condom use, healthcare seeking and health issues. The interview guide was developed in English and translated into two of the local languages spoken languages in that area. All FGDs were recorded with a digital audio-recorder. Data was collected from September 2017 to February 2018.

**Ethical considerations**: the Sefako Makgatho Health Sciences University Research Ethics Committee granted ethical clearance for conducting the study (SMUREC/H/31/2017: PG), and the CPC provided permission for data collection. All the participants provided informed consent before they participated in the focus group discussions. To ensure anonymity, pseudonyms were used for all the participants during the discussions.

**Data analysis**: thematic content analysis was used to identify key themes using the NVivo version 10 qualitative data analysis software. The audio files were listened to repeatedly to immerse the authors in the data. Data were transcribed verbatim and translated into English, and during the translation of the transcripts, the researcher ensured that no meaning was lost. A code list was developed for coding the data into themes. The authors identified and examined the data for emerging themes and sub-themes, which were grouped into similar concepts and contexts for interpretation. The coded themes and sub-themes identified were then discussed and interpreted by the authors to reach consensus. Excerpts from the interview transcripts were coded into themes and sub-themes during the analysis of the data, and these themes and sub-themes were developed through repetitive reading of the transcripts and obtaining the meaning of what was said by the participants in the excerpts.

**Ethics approval and consent to participate**: ethical clearance was granted by Sefako Makgatho Health Sciences University Ethics and Research Committee (Ethical clearance number: SMUREC: SMUREC/H/31/2017: PG). All participants volunteered and gave a written consent to participate in the study.

## Results

**Socio-demographics of the FSWs**: the mean age of the FSWs was 33 years and the age range of 19 to 44 years (Table 1). Most of the FSWs started sex work at an average age of 23 years and had been working for about 9 years. Their mean daily income for sex work serviceswasR425.00. Most of the FSWs had secondary education, were unmarried, had one to three children, they reported to have ever received treatment for STIs, and up took HIV testing (Table 1).

**Table 1:** socio-demographics and health information of female sex workers

Variables	Frequency	Variables	Frequency
**Nationality**		**HIV status**	
South African	38	Known HIV Negative status	15
Non-South African	8	Known HIV Positive status	31
**Educational level**		**Age of sexual debut**	
Primary	2	Less than 18 years	4
Secondary	43	18- 25	27
Tertiary	1	26-30	10
		31-32	3
**Marital Status**		**Years in sex work trade**	
Single	42	1-5 years doing sex work	19
Married	3	6-10 years doing sex work	14
Widowed	1	11-15 years doing sex work	5
		16-20 years doing sex work	8
**Number of life children**		**Last HIV test**	
None	5	3 months ago	10
One	16	6 months ago	4
Two	18	12 months ago	32
Three	7		
**Last STI treatment**			
Never got treated	5		
3 months ago	14		
6 months ago	15		
12 months ago	10		
24 months ago	2		

**Main themes and sub-themes from the data**: themes were developed to ensure that properanalysis of the collected data occurred. Several themes that emerged from the data representboth the positive and negative practices and experiences of condom use between female sex workers and their clients. The following is a list of main themes and sub-themes that emerged from the data (Table 2).

**Table 2:** themes and sub-themes from the data

Main themes	Sub-themes
**Context of condom use in sex work**	Condom use in early sex work
	Condom use over time as a sex worker
	Preferred types of condoms
	Self-efficacy to use condoms
**Condom use negotiation strategies**	Requesting and enforcing the use of condom
	Using the health-risk information to threaten or motivate clients to accept condom use
	Charging more for unprotected sex
	Refusing unprotected sex
**Challenges and predicaments related to doing sex work**	Facing violence and lack of control over place of sex
	Using unsafe tactics to prevent STI transmission
	Ways to avoid loss of work days by hiding blood during menstruation

**Context of condom use in sex work**: the FSW reported that, at the onset of sex work, they knew nothing about condoms, what they looked like, how to use condoms, and also why it is important to use them during transactional sex.

**Condom use in early sex work**: this is how the FSWs reported how they used condoms in the early days of their sex work: *To be honest when I first started as a sex worker I didn´t even know what is a condom, I didn´t know how to use it, what I wanted was to make money, my clients when I started were white men only, when a client wanted a blow job I would do it and later wash my hands, but currently I use a condom except if it´s a hand job (34 years old, HIV+, 15 years´ experience of sex work). When I first started in this job, it was twenty rand per client but most clients gave me fifty rand because they did not want to use a condom (41 years old, HIV+, 18 years´ experience of sex work)*.

**Condom use over time as a sex worker**: sex workers only learned about condoms and the importance of condom use to prevent HIV/STI and pregnancy as they gained experience in the trade.

*When I first started as a sex worker, I would agree if a client refuses to use a condom but now if a client doesn´t want to use a condom I ask him to take his money and leave (27 years old, HIV+, 6 years´ experience of sex work). We use both male and female condoms and the sheet condom [the dental dam]. If a client wants a blowjob then I use a sheet condom (40 years old, HIV+, 16 years experience of sex work)*.

**Preferred types of condoms**: the male condom was the most preferred and the female condom was used as an alternative where a male condom could not be used. Very few participants reported using the female condom.

*Most of the time I use male condoms. I use female condoms if I come across a client who complains that a condom hurts him and he doesn´t have the money to add. I will say to the client, because this one is hurting you I am going to put on this female condom, and you don't have to do anything (24 years old, HIV+, 2 years experience of sex work). I use male condoms. Female condoms, I use them when I am menstruating (37 years old, HIV+, 7 years´ experience of sex work)*.

**Self-efficacy to use condoms**: FSWs had different ways of using condoms with their clients. Some reported using more than one male condom for a sexual act. For negotiating condoms use, some FSWs employed strategies such as enforcement and direct request or indication that the clients will know that the sexual act will involve condom use. The following themes will present self-efficacy of condom use among FSWs and strategies used to negotiate the use of condoms with clients.

*…you can use one condom, but most of the time I use two condoms, the number of condoms you are going to use depends on the type of a client you are with at that moment(37 years old, HIV+, 8 years´ experience). If the client is drunk, he becomes rough, so you ensure that if it happens that the condom burst your are safe because the other one (40 years old, HIV+, 19 years´ experience of sex work)*.

**Condom-use negotiation strategies**: the data revealed four types of condom use negotiating strategies, which were used by the FSW on their clients. The strategies used were requesting and enforcing condom use, using “health-risk information” to threaten or motivate clients to use condoms, charging more for unprotected sex, and refusing unprotected sex.

**Requesting and enforcing the use of a condom**: the data revealed that FSWs would produce a condom and show it or give it to a client for enforcement. In addition, FSWs would ask the client about the number of preferred condoms or whether the client had his own condom.

*I ask for money first and on the other hand, I openly hold and show a condom (40 years old, HIV+, 20 years´ experience of sex work). When we enter the room, I say to a client “do you have a condom?” because sometimes clients when you offer him a condom they will tell you that “don't worry I have my own condom.” But some tear the condom up without you noticing, you will realise by the wetness during sex that the condom is burst…(34 years old, HIV+, 10 years´ experience of sex work)*.

**Using the health-risk information to threaten or motivate clients**: some FSWs reported to use health-risk information as a threat or motivation for the clients to agree to use condoms.

*You are seeing me for the first time and you already want to have sex with me without a condom, while I´m doing high risk work. We FSWs, when it comes to illnesses we are topping the list, we are the ones that are killing people, why don´t you take care of yourself? I value my life, I don´t want to have a relationship with you because I don´t want to see myself sick and die, so please listen to me and let us use condoms...(37 years old, HIV+, 8 years experience of sex work)*.

*“…If you do not want to use a condom you'll end up being infected because I had abscesses that are infectious in the vagina… I have AIDS”. And the client end up putting two condoms (35 years old, HIIV+, 3 years´ experience)*.

**Charging more for unprotected sex**: during the discussion, the FSWs reported that they have different prices for sex with a condom and for sex without a condom, and sometimes clients offer the amount of money they are willing to pay for non-condom use.

*The other cause of this money, the client will offer me two thousand Rands for not using a condom, wouldn´t you take it? You don´t have a choice, you will succumb to the client´s demands (34 years old, HIV+, 8 years´ experience of sex work). I am still not using a condom to certain clients if they offer me five hundred Rands, (40 years old, HIV+, 16 years´ experience)*.

**Refusing unprotected sex**: participants reported that in some instances they refuse unprotected sex with clients even if they offer them more money and are not concerned about losing the money or clients to another sex worker, because they are not willing to put themselves at risk of contracting HIV/STIs.

*When I first started sex work, I would agree if a client refuses to use a condom but now if a client doesn´t want to use a condom I ask him to take his money and leave (27 years old, HIV+, 6 years experience of sex work)*.

*… the client will tell you that his penis can´t get an erection if they put on a condom … I tell them to take their money and leave (30, secondary, HIV+, 5 years experience of sex work)*.

**Challenges and predicaments related to doing sex work**: during the interviews, we asked the female sex workers about the challenges that they are faced with in their daily work The FSW reported that the street-based environment, which is unregulated, exposed them to risks of being attacked by their clients and to lack of access to healthy infrastructure for supporting safe sex such as ablutions. The sex workers reported that they do not have control over their clients´ behaviours, because the sex act is sometimes performed in the bushes, in the clients´ car, in the clients´ own place, sometimes in places rented by clients, and or even in the sex workers´ rooms. It is just the two of them in the room, so most of them are vulnerable to and experience all forms of abuse from their clients.

**Facing violence and lack of control over where to have sex**: the FSW reported that the street-based environment, which is unregulated, exposed them to risks of being attacked by their clients and to lack of access to healthy infrastructure for supporting safe sex such as ablutions. General reports among the FSW were that they experienced violence from their clients due to the street-based nature of their work.

*Sometimes you get a client who is kind to you and take you to the bushes, when you get there he points a gun at you and tell you that you are going to have sex without the condom and you are going to give in to his demands (23 years old, HIV+, 6 years´ experience of sex work). A client will book you for the whole night and take you to his place. He then point a gun at you and forces you to have condomless sex… you end up saying it´s better not to have sex without a condom instead of dying (30 years old, HIV-, 2 years´ experience of sex work)*.

**Using unsafe tactics to prevent transmission of STIs**: female sex workers used various materials, which they insert inside the vagina before unprotected sex (a sponge or cotton wool or a stocking) as a strategy to prevent the semen from coming into contact with the cervix, which is perceived as to be protective against STIs.

*On the streets, there are clients who refuse using condoms. The female condom has a sponge inside. Most of us insert the sponge in the vagina.. When the client asks to urinate (before sex), you insert the sponge and he gives you the one thousand Rands that he has promised you. After he finishes, you take out the sponge (33 years old, HIV+, 5 years´ experience in sex work). … clients have a choice. If a client does not want to use a condom, I insert a sponge (30 years old, HIV+, 4 years´ experience of sex work)*.

**Hiding blood during menstruation to avoid loss of workdays**: female sex workers reported that even during their menstruation they have to work in order to earn a living. They use objects like a sponge, stockings, cotton wool or pills, which they insert into their vaginas to block the menstrual fluids from coming out, and they go on with selling sex as usual.

*If it´s towards month end and I have to have money to pay rent and buy food, and I´m menstruating, I take these new female condoms which have a sponge. I take out that round sponge and put it inside my vagina, then after three clients I take it out, wash it then put it back (40 years old, HIV+, 16 years´ experience of sex work)*.

*In order for the client not to notice that I am menstruating, I usually use strawberry flavoured condoms because the condom is also red in colour (34 years old, HIV+, 15 years´ experience of sex work)*.

*At first when I realised that I´m menstruating I took Aspirin and inserted it inside my vagina and within thirty minutes the blood stops, (41 years old, HIV+, 18 years´ experience of sex work)*.

**Discussion**

This study was conducted among the street-based FSWs in a district of Gauteng Province in South Africa. Almost all the FSWs came from outside Gauteng Province, and have been in the sex work industry for over one year to 20 years long. Most reported that they were HIV positive. The themes that emerged from the data on challenges to negotiation and condom use among FSWs revealed the ways condoms are used in early sex work and over time, ways of enforcing condom used preferred types of condoms and the predicaments to working in the sex trade. The findings show that at the onset of sex work, the FSWs lacked basic health information on condoms and the importance of condom use during sex, and even when they had access to condoms, they did not know how to insert or use them as cited in a review of studies conducted in sub-Saharan Africa [18]. Our research findings demonstrate that condom use skills are learned on the job through interacting with other sex workers as well as through peer-educators doing condom use demonstrations and this is also illustrated in other sub-Saharan studies [8,19]. Peer-education further exposed the FSW to available types and correct use of condoms (male, female and sometimes dental dam) [18].

In this study, male condoms were identified as the preferred type of condom by all sex workers. Although some of them knew how to use the female condom, it was still not their preferred type of a condom and this was also cited in a systematic review of African studies [8]. The female condom was described by the FSWs as something that they used optionally when a male condom could not be used, this suggests that there is a need to rigorously promote and promote female condom use among FSWs, as is advocated in other studies [20,21]. Low uptake of female condom and criticism from the women in the general population including the FSWs has been reported overtime in studies from sub-Saharan Africa [21-23]. The above-mentioned findings raise concern, since they down-play the empowerment that could be derived from using a condom without the need to negotiate its use, which is possible only with the use of a female condom. The findings show frequent reporting from FSWs of clients refusing to use condoms, experiences of violence, physical abuse, and being overpowered by clients with the FSWs ending up having condomless sex as was cited in other studies [24-30]. However, the data revealed that some of the FSWs were quite confident in their condom use strategies and stood their ground in the face of their clients by refusing to have unprotected sex. The findings suggest that the interventions promoting 'no sex without a condom’ such as the FSWs in this study received from the CPC, seem to be empowering the sex workers to refuse unprotected sex with clients. But this does not empower them with the skills to effectively negotiate condom use in all situations as was mentioned in other studies [19,28]. Therefore, going forward, the peer-education interventions provided to the FSWs should not only empower them to refuse unprotected sex but should also build their skills in convincing or persuading unwilling clients to use condoms.

The use of health information messages as scare tactics was found to be successful in enforcing condom use with clients in many instances. This often resulted in clients opting to double-up condoms to prevent transmission of infections. The findings of this study underscore the need to strengthen health promotion programs and education on the safe and effective use of condoms. This in light of FSWs in this study reporting that they use two condoms instead of one as a form of added protection particularly when dealing with rough, drunk, and possible abusive clients. Evidence shows that doubling condoms with the intention of increasing protection from HIV infection is a common practice among FSWs globally [31-35]. We identified four condom use negotiation strategies used by FSWs in this study. The FSWs employed a direct request for condom use by their clients, they used health-risk information, they charged more for unprotected sex, and would sometimes refuse to engage in unprotected sex by letting the client go without buying sex. These strategies were found to be used by FSWs in other studies [14,15,36-39]. The study findings suggest that FSWs have some level of self-efficacy in condom negotiation, which demonstrates that health promotion has taken place and there has been behaviour change. This is particularly true in the enforcement of condom use and the use of health information as both a motivator and a threat to force clients to use condoms. This suggests that some of the FSWs in this study were empowered to a certain extent and wanted to protect themselves and others (their clients and other sexual partners, like their clients´ wives and girlfriends at home). These findings are consistent with the findings of studies conducted on programmes that have been tailor-made for sex workers [37,40,41].

The refusal of unprotected sex also means that the FSWs have been empowered to negotiate condom use, as they are able avoid exposing themselves to STIs for the sake of making more money. These findings are in consensus with those reported in Uganda [42]. However, charging more for unprotected sex is a risky behaviour that still occurs, since some FSWs succumb to non-condom use in order to earn more money [40]. The data show that even experienced female sex workers who know about the health risks involved in non-condom use still abandon condom use negotiation with clients in the interest of raising the price for sex. Despite the reported condom negotiation strategies, FSWs did not have other ways to convince an unwilling client. This was also found in other studies [14,15,36,39,41]. Huschke [28] suggested peer-led risk reduction workshops could benefit the sex workers and empower them for increased use of condoms. Of concern is the use of practices that are unhygienic and risky to their sexual and reproductive health, such as the insertion of materials in the vagina during their menstrual periods for perceived blockage of semen into the cervix and uterus. The reports of rinsing and reusing these materials after sex, forgetting to extract materials at the end of the sex workday is more worrying as it put the FSWs to high risk of infections. Inserting materials such as sponges and cloth in the vagina was also reported by Coetzee *et al*. [25] and other studies reported douching and using substances such as salt, vinegar, lemon and beer to tighten the vagina and or to hide blood during menstruation [43,44]. Insertion of materials and the reported rinse and reuse of the materials constitute gynaecological health hazard for the FSWs as cited in a South African study [2].

Limitations of the study: the findings of this study need to be interpreted with consideration of some limitations.The findings reported are based on self-reports by street-based FSWs around an urban area in the main transit road of Gauteng Province. Due to the sensitive and private nature of the topic, the FSWs were initially not comfortable to speak in groups, but, as the interviews progressed, they became more relaxed and were able to share their personal and sexual experiences about their condom use practices and experiences. This may have introduced social desirability to the data. However, the strength of the study is that the experiences of the FSWs were described from their viewpoints.

## Conclusion

Most of the FSWs in this study reported good understanding of condom use and were confident to use condoms correctly. Of concern is that most of the FSW were introduced to condom use education through peer-education after some time of doing sex work, which put them at risk of acquiring HIV at the onset of their involvement as sex workers. Once educated on HIV prevention and condom use the FSWs became empowered to use and negotiate condoms. Female sex workers´ condom negotiation strategies illustrated that peer-education and sharing experiences among themselves were beneficial for protective sexual behaviours. The benefit of the peer-education and their interactions yielded assertive attitudes and behaviours of demanding and/or enforcing condom use with clients, adapting health-information messages for enforcing condom use, and refusing unprotected sex. The positive protective condom use behaviours that were learned were sometimes flaunted or abandoned when the FSWs needed to make more money because of their financial responsibilities, and when they were violated by the male clients through physical and sexual assault. Over and above charging more for condomless sex, family needs and financial responsibilities called for employing unsafe vaginal hygiene and other risky decisions among the FSWs. Some of the unhygienic and risky practices included inserting materials in the vagina for perceived blockage of semen into the cervix and uterus; rinsing and reuse of the materials after sex; and sometimes forgetting to extract materials at the end of the sex workday. Insertion of materials in the vagina was also used to avoid missing days of work during menstrual periods.

Sexual health intervention programmes, such as HIV peer-education and pre-and post-exposure prophylaxis programmes that are tailored for key populations including the FSWs have shown to be effective with regards to condom use self-efficacy. The FSWs know the health risks associated with non-condom use. Condom use is dependent on both the sex worker and the client or stable partner. There is inconsistent condom use in the FSW industry due to economic factors and the nature of the relationships formed, including the threat of sexual partner violence. This study shows that the effectiveness of condom use interventions are challenged by the socio-economic context of the FSWs, which promotes inconsistent condom use, condom use failure, and unsafe vaginal hygiene practices, and could lead to poor reproductive health outcomes. The findings in this study could help in the development or adaptation of effective health interventions for sex workers. The findings raise ideas on vaginal health education and promoting safe vaginal hygiene practices among FSWs. The given challenges raise questions on development innovative tools that could promote safety and health during sex work.

### What is known about this topic

- FSW carry the highest burden of HIV and STI´s with those based on the street with an increased vulnerability to HIV/STI as compared to those based in establishments;
- FSWs lack power to negotiate condom use, due to their being in unequal relationships with male clients who are their source of income.

### What this study adds

- The effectiveness of condom use interventions are challenged by the socio-economic context of the FSWs
- Peer-education and sharing experiences among FSW is beneficial for protective sexual behaviours. Peer-education benefits and peer-interactions yield assertive attitudes and behaviours of demanding and/or enforcing condom use.
